# Sustainability assessment and causality nexus through ecosystem service accounting: The case of water purification in Europe

**DOI:** 10.1016/j.jenvman.2018.06.072

**Published:** 2018-10-01

**Authors:** Alessandra La Notte, Silvana Dalmazzone

**Affiliations:** a European Commission Joint Research Centre, Directorate D Sustainable Resources, Via Fermi 2749, 21027 Ispra (VA), Italy; b University of Torino, Dipartimento di Economia e Statistica "Cognetti de Martiis", Lungo Dora Siena 100/A, 10153 Torino, Italy

**Keywords:** Ecosystem accounting, Ecosystem services, Water purification, Sustainability assessment, Causality nexus

## Abstract

The paper builds on the Supply and Use Tables module within the System of integrated Environmental and Economic Accounts - Experimental Ecosystem Accounts (SEEA EEA) developed by the UN. We explore the evolution of Supply and Use Tables from the System of National Accounts (SNA) to the System of integrated Environmental and Economic Accounts - Central Framework (SEEA CF) and then to the SEEA EEA, and we propose a further extension: we propose that ecosystem types should be treated as accounting units able to produce, consume and exhibit changes in regeneration and absorption rates. The implications are first explained in the methodological section and then shown in the application where the water purification service is tested against two major policy issues: sustainability assessment (we show how to assess whether the ecosystem service is used sustainably by comparing the quantification of potential and actual flow) and causality nexus (we quantify the connection between the value of agricultural production and that of the ecosystem service used). The paper highlights how the overall outcomes change when considering different scales. A contrast emerges, for example, between the positive balance at the continental scale, where water purification services appear to be used sustainably (thanks to the high potential flow of Northern European countries) and the negative balance of almost all European countries when considered at a national scale. Taking advantage of the experimental opportunities offered by operating with external satellite accounts, we are able to show how the proposed complementary tables could support policy action.

## Highlights

• Ecosystem services accounts are quantified through supply and use tables.
• In order to assess sustainability we need to report both potential and actual flows.
• In order to track a causality nexus we need to separate services from benefits.
• We also need to separate enabling actors from beneficiaries.

## 1 Introduction

A separate analysis of the economy on the one hand and of ecosystem services on the other does not adequately reflect the fundamental relationship between humans and the environment. Only the integration of ecosystem and economic information would allow mainstreaming evidence on ecosystems and their services within public and private decision making (UN et al., 2015). Accounting systems enable the organization of information in an integrated and conceptually coherent manner. This information can then be employed to create scientifically rigorous indicators to be used to inform environmental management and policy choices.

Economic information is provided by the *System of Nationals Accounts* (SNA), a measurement framework that has been evolving since the 1950s to measure economic activity, economic wealth and the general structure of the economy. The strength of the SNA is its robust articulation that allows for a certain deal of flexibility while still remaining integrated, internally consistent and economically complete. However, the SNA framework should neither be overburdened with details nor containing conflicting requirements, likely occurring when a different representation of the economic process leads to different aggregates.

Recognizing a need for flexibility, since its 1993 version (UN et al., 1993) the SNA incorporated the concept of satellite accounts – additional accounts closely linked to the main SNA but not restricted to the same concepts and data. There are two types of satellite accounts: (i) *internal* satellite accounts follow entirely the accounting rules and conventions of the SNA but focus on a particular aspect of interest (tourism satellite accounts are an example); (ii) *external* satellite accounts may add non-economic data and/or vary some of the accounting conventions (e.g. adopt a different production boundary, or consider additional assets). External satellite accounts allow experimenting with new concepts and methodologies in a research context with much wider degrees of freedom (EC et al., 2009).

The System of integrated Environmental and Economic Accounts (SEEA) is a set of satellite accounts; it applies the accounting concepts, structures, rules and principles of the SNA to environmental and natural resources in order to integrate environmental information (often measured in physical terms) with economic information (generally measured in monetary terms) in a single framework. The SEEA- Central Framework (SEEA CF) embeds both internal (e.g. environmental protection expenditures) and external (e.g. non-produced environmental assets) satellite accounts. The SEEA CF provides guidance on the valuation of renewable and non-renewable natural resources as well as land within the asset boundary of the SNA (UN et al., 2014a).

In the SEEA CF a distinction is made between the measurement of environmental assets as individual natural resources, cultivated biological resources, land, and the measurement of environmental assets as ecosystem components. A platform for the integration of relevant information on ecosystems and ecosystem services has been recently proposed and supported by the United Nations Statistical Division (UNSD): the System for Integrated Environmental and Economic Accounting-Experimental Ecosystem Accounting (SEEA EEA) (UN et al., 2014a, b; UN et al., 2014b, UN et al., 2017). The SEEA EEA is designed to facilitate comparison and integration with the economic data prepared following the SNA. Specifically, ecosystem information is presented together with standard measures of income, production and wealth. The SEEA EEA is meant for application at a national level (as the SNA) in order to link information on multiple ecosystem types and services with aggregate economic and planning decisions. The SEEA EEA includes only external (ecosystem and ecosystem services) satellite accounts.

In this paper, we adopt an innovative perspective that includes ecosystem as units playing an active role in an economic context. Concepts and structures from other disciplines (environmental science, hydrology, forestry, fisheries, economics, statistics) need in fact to be accommodated within the accounting method, while preserving the SNA as the underlying structure. In our case, we are going to consider ecosystem units providing service flows as institutional sectors and thus to account for what happens to ecosystem units while interacting with economic units. What we want to avoid is to consider ecosystem units merely as providers of inputs to economic sectors and households.

A few previous applications of this perspective have been proposed, most of them at a local scale (La Notte et al., 2011; Busch et al., 2012; Schröter et al., 2014; Remme et al., 2014) and one for Europe (La Notte et al. 2017a, b). All existing studies address methodological and conceptual issues related to the ecosystem accounting procedure and practice. This paper has a more theoretical objective. It aims at exploring in depth and testing the connection between ecosystem services and economic accounts. Specifically, we build on the water purification case studied by La Notte et al. (2017a, b) and, working on biophysical data disaggregated at the national level for 34 countries, we show how they link with national accounts and what information can be obtained by connecting ecological and economic information.

The following section (§2) briefly introduces the main components of the SEEA EEA and focuses on Supply-Use tables, which represent the reference tool for our application. In section 3 the case of water purification and its linkages with economic accounts is presented and explained. In section 4 we demonstrate the critical role played by the scale of analysis in the interpretation of the information provided by environmental accounts. Section 5 offers discussion and conclusions.

## 2 Integrating ecosystem services and economic accounting: the methodological frontier

**Fig. 1:**
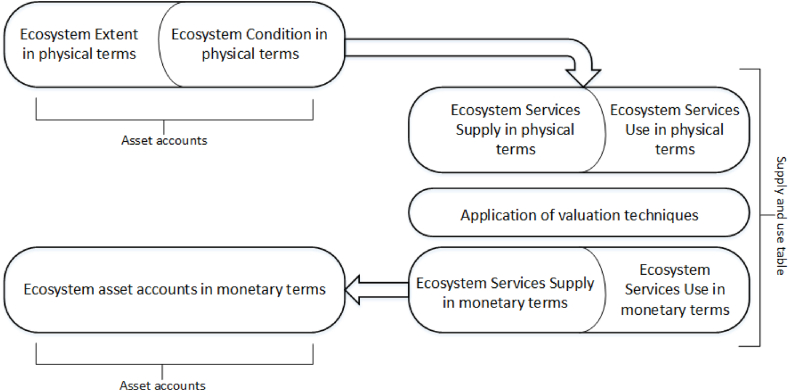
Components of the SEEA EEA (adapted from KIP-INCA Phase 1 report, 2016).

The SEEA EEA is composed by two sets of accounts: (i) *ecosystem asset accounts*, quantified through the ecosystem extent account and the ecosystem condition account, and (ii) *ecosystem service accounts,* quantified through Supply and Use tables to be quantified in both physical and monetary terms (Fig. 1). The ecosystem asset account in monetary terms can be compiled by aggregating the Net Present Value (NPV) from ecosystem services Supply and Use tables. An important element in our analysis is that Supply and Use tables are the accounts with a direct connection to economic sectors.

According to SEEA EEA definitions, the stocks in ecosystem accounting are represented by spatial areas, which constitute an ecosystem asset. Flows in ecosystem accounting are of two types: (i) flows within and between ecosystem assets which reflect on-going ecosystem processes (e.g. intra- and inter-ecosystem flows); (ii) flows generated by ecosystem assets and directed to people, i.e. ecosystem services. Flows of ecosystem services may relate either to flows of natural inputs from the environment to the economy or to flows of residuals from the economy to the environment. Ecosystem services provide the link between ecosystem assets and the benefits derived and enjoyed by people. In the SEEA EEA ecosystem services are not ecosystems and are not benefits; they are ecological processes connecting the two.

The SEEA EEA focuses on external (ecosystem and ecosystem services) satellite accounts, whose experimental perspective allows using the SNA articulation to frame, in a consistent economic context, an enlarged production and asset boundary that includes ecosystem units as playing an active role.

### 2.1 Supply and use tables: from the SNA to the SEEA CF

In the SNA, Supply and Use tables describe the structure of the economy and the level of economic activity by recording all flows of products between different economic units in monetary terms. The Supply table provides records on what is domestically produced by industries and what is imported from the rest of the world. The Use table provides records on the intermediate consumption by other industries, final consumption by households and government, exports and what is not consumed in the current period. The latter includes (i) changes in inventories (additions to inventories less withdrawals), and (ii) changes in fixed capital (e.g. machinery used over a longer period of time to produce other products). Changes in inventories and in fixed capital are recorded as “accumulation”.

All flows are classified by type of product in the rows and by institutional sector (enterprises, households, government and the rest of the world) in the columns. Enterprises are identified on the basis of their principal activity. Institutional sectors are grouped together on the basis of similar objectives, purposes and behaviour. The accounting identity that must be fulfilled is that Supply must equal Use.

**Fig. 2:**
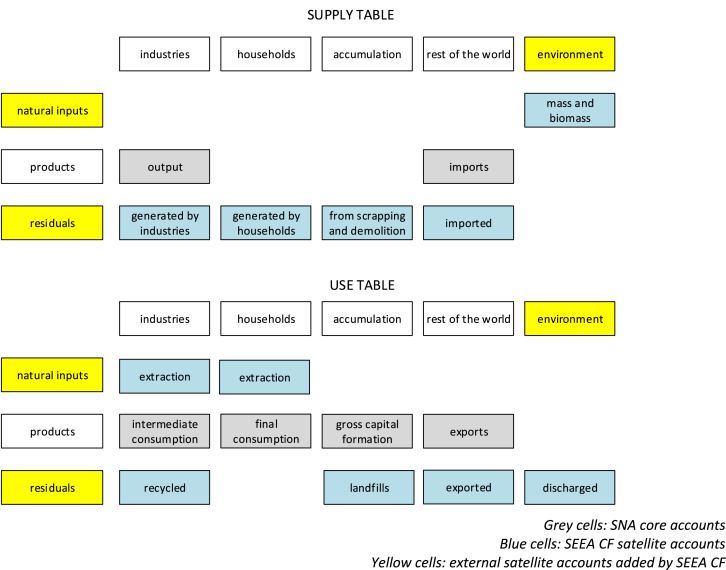
Graphical simplification of monetary Supply and Use table in the SEEA CF (source: adapted from La Notte et al., 2017b).

Economy and society withdraw flows of mass and biomass from the environment and discharge flows of residuals to the environment. In the SEEA CF Supply and Use tables record, in physical and monetary units, flows of natural inputs and residuals. External satellite accounts are added to the SNA accounts in terms of source of natural inputs and destination of residuals (the column “environment”) and in terms of additional flows to be recorded (the rows “natural resources” and “residuals”). Fig. 2 shows how the SEEA CF complements the SNA for Supply and Use tables.

Although the inclusion of the column “environment” makes it possible to fully account for flows of natural inputs and residuals, the environment in the SEEA CF still remains a passive entity, since its production, consumption and changes in functioning are not recorded (UN et al., 2014a).

### 2.2 Supply and use tables in the SEEA EEA

In the SEEA EEA the external satellite account concerning the environment stops being purely passive. It is structured according to ecosystem types (e.g. arable land, natural grassland, wetlands, woodland and forests, rivers and lakes^1^) and it provides a series of ecosystem service flows (if recorded according to the CICES classification they would be grouped in ‘provisioning’, ‘regulating and maintenance’, and ‘cultural’) that constitute the rows of the new Supply and Use tables. The flows recorded in the SEEA EEA Supply and Use tables are the actual flows of ecosystem services in terms of transactions or exchanges that take place between ecosystem types and economic sectors and households.

**Fig. 3:**
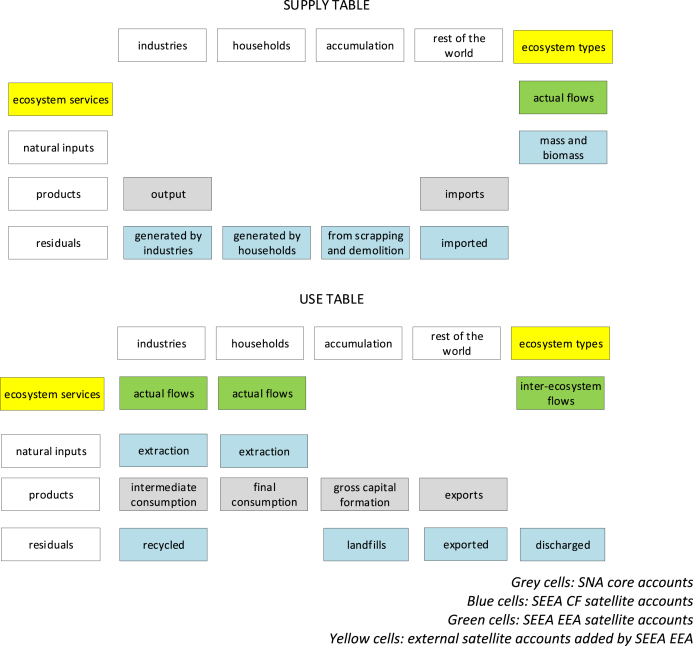
Graphical simplification of Supply and Use tables in the SEEA EEA (source: adapted from La Notte et al., 2017b).

In Fig. 3 we show a combined presentation of SEEA EEA, SNA and SEEA CF Supply and Use tables. The purpose of having a combined presentation is to clearly visualize that the flow of ecosystem service differs from the flow of benefits, which may be SNA benefits or non-SNA benefits. Some SNA benefits (especially those generated by provisioning services) are likely to coincide with SEEA CF natural inputs. The SEEA EEA Technical Recommendations (SEEA EEA TR, UN et al., 2017) specify that in the Use table it is possible to record the use of ecosystem services by other ecosystem types.

The SEEA EEA structure for Supply and Use tables described above is indeed the ideal starting point to develop pilot applications. One particular aspect we would like to stress is that the SEEA EEA TR explicitly mention that the measurement scope of ecosystem services in the SEEA EEA is defined in the context of the SNA production boundary: a few important elements are added, while the production boundary remains the same. Although conceptually possible, the option to alter the production boundary of the SNA has not been fully explored yet. This is precisely what our study investigates: we use the opportunity offered by experimental external satellite accounts to attribute to the environment the ability to play a full active role as it happens for economic sectors.

Production, consumption and accumulation by economic sectors are recorded and consistently framed in the accounting system. By becoming an institutional sector (in accounting terms), ecosystem types should be able to see recorded not only their provision to economic units but also their full interaction with economic units. For ecosystem services it is not feasible to account for accumulation (“physical mass and biomass” is a benefit, ref. Fig. 2, Fig. 3 “Supply table”). In fact, we should keep in mind that we are quantifying ecological processes: what flows into the “fixed capital” (i.e. accumulation) is the capacity of the ecosystem to provide a certain amount of a service flow for the next period. For ecosystem services it is relevant to represent changes in regeneration and absorption rates that will affect future provision of the service flow.

**Fig. 4:**
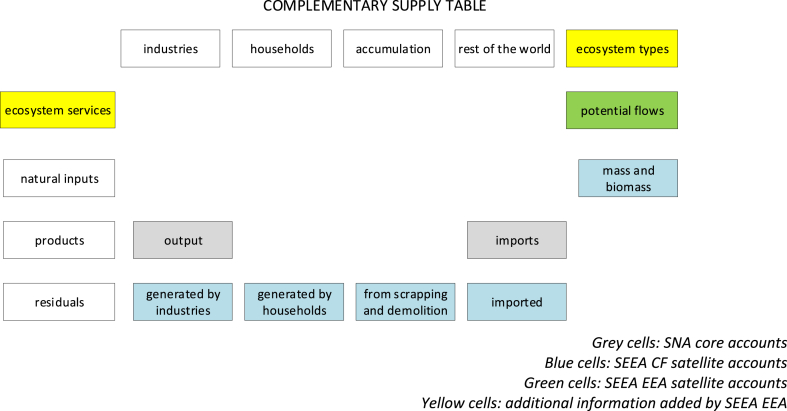
Graphical simplification of the complementary Supply table to be added to the SEEA EEA.

When an ecosystem service (provided by the relevant ecosystem units) is identified because there is a human need for it, two different kinds of flows should be reported in accounting: (i) on the one hand, we have the total flow that relevant ecosystem types are able to generate for each individual service (*potential flow*); (ii) on the other hand, we have the amount that is currently used by economic sectors and households, which we can call *actual flow*. In addition to the standard Supply table reporting actual flow, we can compile a complementary Supply table that reports the potential flow, that is what ecosystem types are able to offer, independently of how much of it will be used by economic activities (Fig. 4). The complementary Supply table thus allows us to record the production stage.

The Use table makes it possible to quantify consumption (in terms of inter-ecosystem flows) and changes in ecosystem service regeneration and absorption rates. The latter are calculated as the difference between potential and actual flow. When, for example, actual flow is higher than potential flow an overuse of the ecosystem service occurs: in this context, the accounting concept of depreciation (of fixed capital) translates in accounting terms the ecological concept of overuse that eventually leads to degradation. Thus ecosystem types in the Use table allow us to account for ecosystem depreciation (or appreciation) when the sign of the difference between potential and actual flow is negative (or positive).

By proposing a combined presentation, we have the opportunity to visualize the role and place of SNA and non-SNA benefits. The latter also include intangible benefits such as those accruing to individuals as a result of living in a clean environment, cultural values and any other changes in the level of human wellbeing unrelated to the direct consumption of products that can be exchanged on the market (such as food, water, shelter, clothing, recreation). Non-SNA benefits are currently not covered by any accounting framework.

The importance of clearly separating the service flow generated by ecosystem units from the final benefit perceived arises specifically in cases where those who activate the service (enabling actors) differ from those who perceive the final benefit (beneficiaries). This is true especially for sink-related services. Consider the case of pollution (e.g. air filtration, water purification, soil decontamination). We can distinguish between upstream actors and downstream beneficiaries. Upstream actors are those who create the need for the service, without whom the service would not be there, and who have the possibility to modify the actual amount of the service flow. The perceivers of SNA and non-SNA benefits are the agents who receive the outcome of what is generated by the service: households and economic sectors to whom the benefits in terms of clean air, clean water, etc. will be attributed. By allocating the flow of the service to upstream actors we can establish the causality nexus between the behaviour of economic actors and the effects on ecosystem types. When services and benefits are embedded it is not possible to establish a link between who modifies the actual flow and who enjoys or suffers the effects of this modification. When services and benefits are disentangled, policies are able to address the actors who have the power to modify the actual flow of the service, because the causality between them and the impact in terms of generation of benefits and flows returning to ecosystem types can be identified (La Notte and Marques 2017).

## 3 An experimental application of Supply-Use tables to water purification

**Fig. 5:**
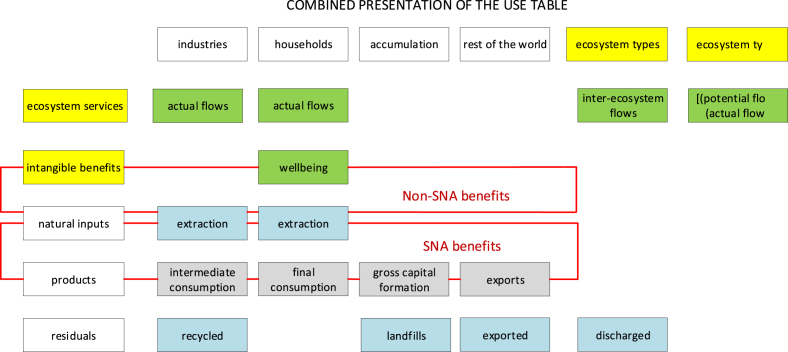
Graphical simplification of the Use table supported by complementary information to be added to the SEEA EEA.

**Fig. 6:**
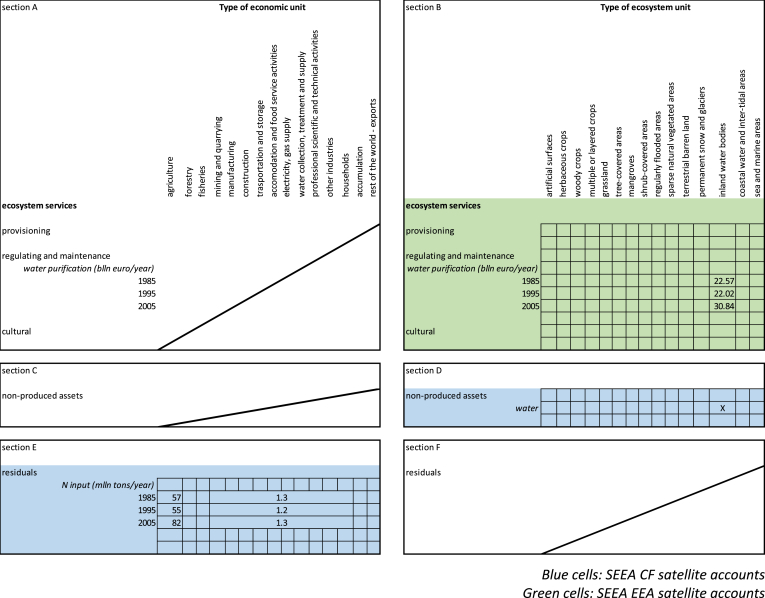
Combined presentation of complementary Supply table for water purification for 34 European countries.

In this section we apply the structure developed in Section 2 and presented in Fig. 5, Fig. 6 to the case of water purification. The example will also help clarifying the rationale of allocating benefits from ecosystem services to upstream actors, as withdrawers of the service.

A previous publication (La Notte et al. 2017a, b) presented in detail the case of ecosystem service accounting for water purification and the steps behind the assessment in physical units and the valuation in monetary terms. The GREEN (Geospatial Regression Equation for European Nutrient losses) model contains a spatial description at a European scale of nutrient sources and physical characteristics influencing nutrient retention (Bouraoui et al., 2009). For each sub-catchment, the model considers the input of nutrients from diffuse and point sources and estimates the nutrient fraction retained during the transport from land to surface water (Basin Retention) and the nutrient fraction retained in the river segment (River Retention). In the case of nitrogen pollution, diffuse sources include mainly mineral fertilizers, manure applications and crop fixation, while point sources consist of industrial and wastewater treatment discharges. For this reason, we consider diffuse sources as representing the ‘agriculture’ sector and point sources representing ‘other industries and households’. Damages from diffuse source pollution are reduced both by processes occurring on the land (crop uptake, denitrification and soil storage), and by those occurring in water systems (aquatic plant and microorganism uptake, sedimentation and denitrification). Point source pollution, instead, is considered to reach surface waters directly, and hence can be mitigated only by river retention. The ecosystem unit is thus ‘inland waters’. Nitrogen retention is computed on an annual basis.

As described in the previous section, we calculate not only the actual flow but also the potential flow by establishing a sustainability threshold: to be sustainable, the removal of nitrogen by the different ecological processes must remain below the critical threshold level.^2^ In the pilot application to which we refer (La Notte et al. 2017a, b) the sustainability threshold was constant for simplicity. However this threshold should indeed ideally differ depending on sub-catchment location (upstream versus downstream) and according to countries.

When the nitrogen input into a river increases, total nitrogen retention may increase as well. If one measured only the total nitrogen retention at a given location (i.e. the actual flow), higher retention would lead us to record an increased flow of the water purification service, which would fail to account for the overuse of the ecosystem service. To address this issue, we assess the value of the provided ecosystem service with reference to the relative position of the actual flow with respect to the sustainability level (i.e. the potential flow). Different *ad hoc* thresholds would generate more precise results. We would however expect the overall trend to remain the same (i.e. a lower level of N would imply a higher potential flow and a lower actual flow, and *vice versa*).

Following the methodological approach described in La Notte et al. (2015), the valuation of the ecosystem service is led by the biophysical assessment; the assessed changes over time are then translated in monetary terms. In the case of water purification, the valuation process consists of three steps. (i) First, a biophysical assessment of the amount of nitrogen retained and removed by rivers and lakes is conducted. (ii) The result is then converted into a *Constructed Wetland Equivalent Area* (CWEA), which is a spatial measure of the ecosystem capacity to provide the water purification ecosystem service. The CWEA provides an estimate of the total area (in hectares) of a wetland extension that would be necessary to provide in each sub-catchment the same nitrogen retention as the river network. (iii) We then use a Replacement Cost approach to estimate the monetary value of the physical units of nitrogen removal produced by the CWEA. For the monetary valuation we combined the economy of scale effect with production costs, differentiated by country. Labour costs were extracted from the Eurostat labour statistics, while costs of filling materials were obtained through a direct survey conducted among CW designers and builders in different European countries. Discounting has been implemented according to the SEEA CF guidelines (Annex A5.2 in UN. et al., 2014a).

Further details (including the rationale behind the choice of using replacement costs) are available in previous publications (La Notte et al. 2017a, b and La Notte et al., 2012a,b). The output is an indicator consistent with the notion of exchange value defined in the SEEA EEA (UN. et al., 2014b and UN et al., 2017). There is in fact a need to use estimates consistent with SNA transaction prices otherwise no comparison between economic and ecosystem services accounts would be possible.

In the result section we report complementary Supply tables reporting the potential flows from the providing ecosystem types; Use tables reporting actual flows allocated to enabling actors; and the difference between potential and actual flows allocated to the providing ecosystem types.

## 4 Results

In this section we are going to present complementary Supply and Use tables, and how they relate with emission accounts and with economic value added figures.

**Fig. 7:**
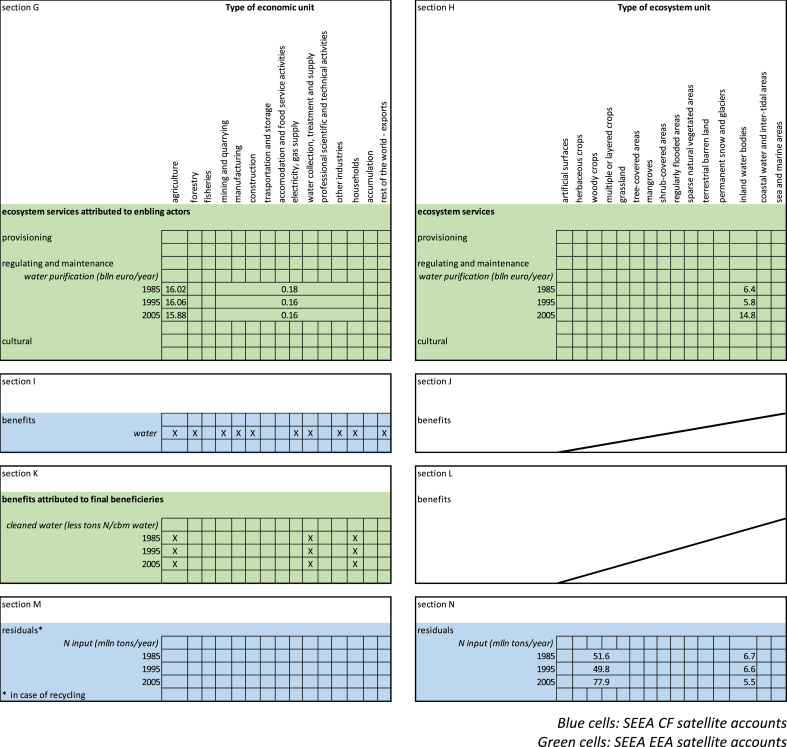
Combined presentation of Use table supported by complementary information for water purification in 34 European countries.

A very interesting point is that the scale at which results are considered is not neutral. Our exercise, by reporting results aggregated at the European and at the national scales, highlights that the choice of scale enables to look at ecosystem services from different perspectives and obtain additional information. Fig. 6 shows the complementary Supply table and Fig. 7 shows the Use table in a combined presentation for water purification at a continental scale. Fig. 6 provides an estimate of how much water purification European inland waterbodies can provide to the economies [section B]; Fig. 7 provides an estimate of how much water purification European economic sectors and households are absorbing [section G]. Here we consider the upstream enabling actors as the service withdrawers, i.e. the primary sector for diffuse sources and industrial processing and households as point sources. Downstream users of clean water have been here supposed to be water supply companies that collect freshwater for distribution.^3^

In both figures we report sections on non-produced assets [section D], benefits [section I and K] and residuals [sections E and N] in order to highlight the linkages with already existing accounts. We report here only the clean-up service performed by inland waterbodies (no other service nor ecosystem type is assessed and valued). As can be seen in Fig. 7, most of the nitrogen is retained in soils, and that process is not part of the present assessment. This should be kept in mind in reading the results: here we are looking at one specific component of freshwater ecosystems, and not at the ecosystem as a whole, and we are considering only one ecosystem service – water purification – rather than the multiple ecosystem services provided by freshwater inland bodies.

At the continental scale supply turns out to be larger than use: ecosystem service accounting shows that at the European level nitrogen emissions in water are managed sustainably. In Europe, total nitrogen emissions to river basins varied between 50 and 80 million tons from 1985 to 2005, the largest share of which (between 80% and 90%) enters the basin from diffuse sources. After basin retention (i.e. the nitrogen that is retained in soils and groundwater), around 5 million tons percolate to the river network. Nitrogen emissions from other economic activities and households enter the river network from point sources and amount to between 1.1 and 1.3 million tons.

The environmental asset more closely related to water purification is surface freshwater. Data related to actual flow of water [section D] and gross total abstraction [section I] can be obtained from Eurostat datasets.^4^ We here only tick where the data should be filled in, keeping in mind that we are not dealing with water provision but with water purification. The reason to look at the water asset is justified by the non-SNA benefit generated by water purification, i.e. clean water [section K]. Clean water should in fact be calculated, in this context, as the fraction of nitrogen-cleaned freshwater abstracted by water supply companies. At the moment, we do not have this indicator: what matters here, however, is showing where values (the indicator of clean water pertaining the outcome of the biophysical model [nitrogen removed] and the water abstracted by water companies) should be allocated and to whom (the beneficiary: water supply companies).

Fig. 7 shows a hybrid account frame, where services are expressed in monetary terms [sections B, G and H] whereas benefits [sections I and K] and residuals [sections E and N] are expressed in physical terms.^5^ Ecosystem services can be reported in both physical and monetary terms. The reason to report them here in monetary terms is the need to link these values with the economic accounts. Non-produced assets [section D] and benefits [section I] can be reported both in physical and monetary terms; benefits [section K] and removals are reported in physical terms. The reason to report here water abstraction in physical terms is to show what part of this information (Mln cbm of water) will be linked to an indicator related to clean water (less N tons/cbm of abstracted freshwater) [section K]. Not all economic sectors that directly withdraw water [section I] need water to be clean [section K].

When moving from the continental scale to the national scale, perspectives significantly change: only three countries (Finland, Sweden and Norway) record potential flows higher than the actual flows. In all other countries this difference is negative (Table 1). To analyse the results more closely at the national level, we selected four countries representative of four different European macro-regions,^6^ namely Denmark, Italy, Germany and Bulgaria.

**Table 1:** Potential and actual flow at the national level.

Country	[(potential flow)-(actual flow)] (euro/km)		
	1985	1995	2005
Albania	−22,386.71	−24,950.88	−28,029.52
Andorra	−20,960.71	−25,657.40	−28,838.37
Austria	−7885.28	−7755.81	−6008.53
Belgium	−44,072.38	−42,423.26	−41,319.79
Bosnia and Herzegovina	−16,658.03	−15,590.13	−16,324.07
Bulgaria	−9373.47	−10,516.53	−11,054.22
Croatia	−14,404.94	−13,797.15	−13,204.12
Cyprus	−5872.32	−5468.48	−5594.36
Czech Republic	−5114.93	426.05	2681.89
Denmark	−60,580.12	−60,214.79	−60,147.62
Estonia	−7358.98	−28,617.27	5634.40
Finland	104,005.52	81,271.44	132,275.08
France	−36,760.73	−39,677.56	−36,471.70
Germany	−11,839.88	−11,338.26	1789.70
Greece	−25,083.49	−25,060.70	−24,746.12
Hungary	−5131.74	−5008.94	−4991.59
Ireland	−18,021.83	−20,807.21	−15,758.13
Italy	−36,671.10	−37,398.57	−35,287.73
Latvia	−36,494.54	−26,184.32	−16,756.48
Lithuania	−32,421.81	−28,831.14	−28,562.70
Luxembourg	−50,043.40	−50,615.11	−47,739.04
Macedonia	−28,780.98	−30,975.22	−30,585.53
Netherlands	−34,537.35	−34,106.92	−33,472.30
Norway	250,550.47	223,791.49	321,860.30
Poland	−3763.81	−1844.36	292.38
Portugal	−14,785.14	−14,680.98	−8402.93
Romania	−5130.45	−4742.98	−5362.37
Serbia and Montenegro	−9168.78	−9948.58	−11,743.35
Slovakia	−7593.43	−6589.67	−6531.76
Slovenia	−18,230.79	−13,760.01	−13,018.01
Spain	−14,969.67	−14,639.44	−13,759.12
Sweden	128,747.55	143,725.82	264,133.60
Switzerland	−31,547.86	−37,616.50	−10,626.92
United Kingdom	−26,139.81	−24,179.70	−24,047.42

Two policy issues are now explored by means of the ecosystem services accounts we have compiled for water purification: (i) sustainability assessment, and (ii) causality nexus with economic accounts.

Let us first conduct a sustainability assessment. This is done by relating pollutant emissions to the potential flow of water purification (calculated in relation to the sustainability threshold). In order to protect water quality from ground and surface water pollution by agricultural sources, the Nitrates Directive was issued in 1991 as part of the Water Framework Directive It represents one of the main instruments for the protection of waters against agricultural pressures and is most likely behind the remarkable decrease in N emissions registered in the European Union (although not in all the 34 countries).^7^

**Fig. 8:**
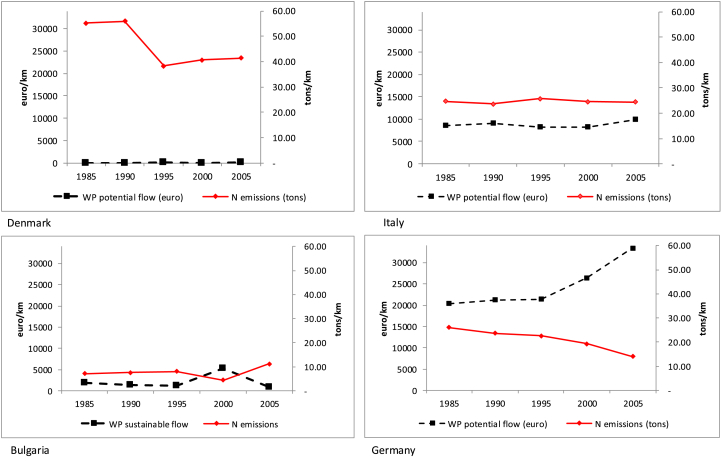
N emissions (tons/km) and water purification potential flow (euro/km).

**Fig. 9:**
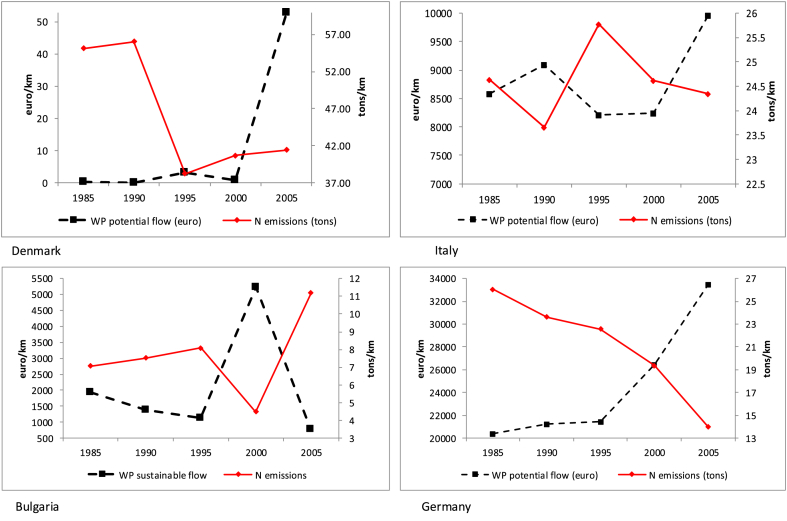
N emissions (1000 tons) and water purification sustainable flow (mln euro) with *ad hoc* scales.

We consider relative emission values, expressed per kilometre of river extend, in order to avoid misinterpretations due to the size of the country. The same principle is applied for the potential flow. Considering a common scale for all countries enables comparisons and the visualization of the overall level of (un-) sustainability (Fig. 8). However, in order to check whether similar patterns occur between emissions and changes in the ecosystem service sustainable flow, *ad-hoc* scales should be adopted (Fig. 9). Here the connection between nitrogen emissions and sustainable flows is evident: the higher the pollutant emissions the lower the potential flow, and *vice versa*. This relationship works only with reference to the potential flow, because the actual flow follows the same trend of nitrogen emissions: the higher the emissions the higher the actual flow, the lower the emission the lower the actual flow.

In countries such as Denmark, the level of N emissions is very high and since degradation is already in place no enhancement is visible when adopting a common scale (Fig. 8). Only considering a different order of magnitude for the potential flow (Fig. 9) makes it is possible to check whether the reduction in N emissions has been generating a more sustainable pattern. In the case of Denmark, reaching sustainability appeared, in 2005, a long term target, but at least the path was heading in the right direction.

The case of Bulgaria is peculiar. Like Denmark, Bulgaria does not record a remarkable service flow (Fig. 8), but differently from Denmark it does not start from a high degradation. By using a common scale (Fig. 8) it is already possible to track a common trend between N emissions and potential flow; however it is when considering an *ad hoc* scale (Fig. 9) that the effect of N emissions on the potential flow is made evident. In the case of Bulgaria, emissions seem to be rising in 2005 and, as a consequence, the potential flow is decreasing.

In Italy and Germany the level of potential flow is much higher than in the previous examples and this is clearly shown when considering a common scale (Fig. 8). This allows us to track already in Fig. 8 a common trend between N emissions and the value of the potential flow: very clear in the case of Germany, more confused and fluctuating in the case of Italy. Germany is indeed on a sustainable path and this is confirmed when looking more in details at the *ad hoc* scale (Fig. 9). In Italy the decrease in N emissions is not a continuous path: this fluctuation makes the effect on the potential flow very unstable, as can be clearly seen in Fig. 9.

A second policy issue that we can explore, at the national scale of analysis, is the causality nexus between economic activities and ecosystem services. Establishing a causality nexus between human activities (i.e. economic sectors and households) and ecosystem services is indeed one of the primary objectives of the SEEA EEA. Specifically, ecosystem service accounts should be able to provide information about how much specific human action affects past, present and future flow of services. This is the main piece of information that is still missing from both the SNA and the SEEA CF.

**Fig. 10:**
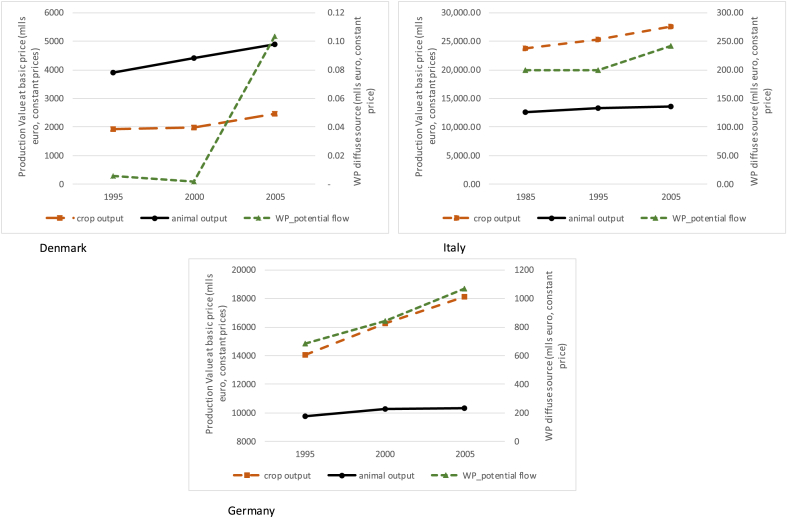
Crop and animal output compared with water purification potential flow (mlln euro).

The first step is properly identifying the sectors that use water purification as upstream enabling actors. For the four selected countries agriculture alone is responsible for almost 90% of nitrogen emissions, with the other sectors accounting for the remaining 10%. For the sake of simplicity, we therefore leave out point sources attributable to industry, tertiary and households and only consider diffuse sources linked to agriculture. In the case of Denmark, Italy and Germany, it is possible to retrieve data concerning crop output and animal output from the economic accounts for agriculture^8^ for the three selected years (1985, 1995, 2005). Fig. 10 reports these economic figures in relation to the trend of water purification, expressed with a different scale. We must keep in mind that water purification is only one, out of at least eight, of the ecosystem services connected to agriculture.^9^ Moreover, what is accounted for here is only the fraction of nitrogen that flows to instream water bodies: less than 10% of the total nitrogen emitted, most of which remains in soil. Fig. 10 should thus not be interpreted as a competition between the relative value of economic activities and that of ecosystem services (at the moment we would not even have, on the ecosystem service side, all the elements to enable this kind of comparison). The intent is rather to acquire some information on the role of economic activities in managing the ecosystem service, on the trends over time, and on the causality nexus between human action and the assessed flow of ecosystem services.

By reading Fig. 10 together with Fig. 8, Fig. 9, we can acknowledge that the fact that no dramatic decrease occurred in animal output and some increase occurred in crop output means that the agricultural sector is implementing more sustainable practices. N emissions decrease, the potential flow increases, and economic figures increase: this is the ecological-economic sustainable path that policies should monitor and pursue.

**Fig. 11:**
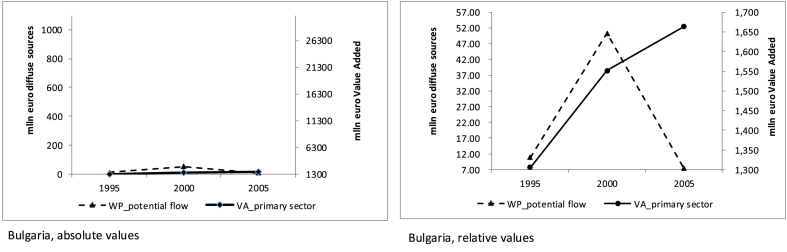
Primary sector Value Added and water purification potential flow (mln euro).

About Bulgaria, for the three selected years (1985, 1995, 2005) we can only report data on the primary sector^10^ Value Added, being aware of the limitation that using this particular figure implies (Fig. 11).

At the beginning of the observation period the Value Added (VA) of primary activities is low; as agriculture develops, also the water purification service is activated. Until the year 2000 the ecosystem service appears to be subject to a sustainable use. However, as the primary sector continues to grow, beyond the sustainability threshold, the estimated value of the potential flow for the water purification service falls abruptly. The case of Bulgaria illustrates that it is not agriculture itself that hinders nature's water purification capacity, but rather its patterns of development. Management practices that reduce the amount of fertilisers in crops and dispose of livestock manure sustainably are likely to make agriculture compatible with the preservation of the water purification sustainable flow. This kind of information would enable policy makers to monitor traditional economic variables such as VA jointly with the related ecosystem service.

## 5 Discussion and conclusions

The main contribution of this paper to the methodological frontier of ecosystem service accounting is to fully exploit the opportunity of conceptual variations offered by using external satellite accounts: not only industries and households are playing an active role in economic production processes but also the ecosystem units in actively delivering a flow of services.

We test this proposal by compiling Supply and Use tables for water purification in Europe, at different scales. The first result we highlight is the contrast between a positive balance at the European scale, where the water purification services offered by natural ecosystems appear to be used sustainably (thanks to the high potential flow of Northern European countries that counterbalances the unsustainability recorded elsewhere) and the negative balance of almost all European countries (Denmark included) when considered at a national scale.

At the conceptual level, considering not only the actual flow but also the potential flow of ecosystem services has enabled us to address two issues of high policy significance: sustainability assessment and the causality nexus.

By comparing potential flow and actual flow it is possible to assess whether an overuse of the service is taking place. Whenever the sign is negative, overuse eventually leading to degradation is occurring. Degradation is going to affect the capacity of ecosystem units providing that specific service to generate the same amount of that service for the future. Sustainability enters into ecosystem service accounting through the assessment and quantification of ecosystem overuse.

The general view is that overuse and degradation should be assessed within the ecosystem condition account. However, a crucial element disappears when moving from individual ecosystem services to ecosystems considered as a whole: the causality nexus between the driver of environmental change and the change itself. This is the reason why we believe it is important to assess overuse also for each individual ecosystem service. The causality nexus is important for policy makers, analysts and practitioners who handle national accounts: monetary variables that matter from the economic perspective should be complemented with estimates concerning ecosystem services. In our exercise, agricultural economic figures and the potential flow of water purification are considered jointly in order to check whether pieces of legislation (such as the 1991 Nitrate Directive) are having an impact on the economy and which kind of impact (i.e. an overall contraction of the sector or the adoption of sustainable practices?). The data from ecosystem service Supply and Use tables can be the starting point for a series of analyses, from the sectorial to the macroeconomic level: the one presented here is only the simplest, basic use of that information. It should be clear that assessing the sustainability of the economic system as a whole is beyond the scope of this paper. The purpose of this work is simply to show the potential direct applications, without any further processing, of ecosystem services accounts.

Ecosystem service accounting is still at a stage where learning happens mostly by doing: the complexity of ecosystem services cannot be conceptualized if not underpinned by concrete case studies, engaging the challenges raised by each ecosystem service with its own peculiarities. Many more analyses will be necessary before environmental management and policy making can rely on a full conceptual and informational basis on ecosystem services. The application presented in this paper can represent, we believe, a small stepping stone along that path.

## Footnotes

1 For more details, see Figure 3.6 in La Notte et al. (2017b).

2 The potential flow was calculated using a sustainability threshold of 1 mg of N/l to avoid ecosystem degradation. This threshold is based on eutrophication risk: levels of total nitrogen lower than 0.5–1.0 mg l-1 could prevent aquatic ecosystems from developing acidification and eutrophication (Camargo and Alonso, 2006; Grizzetti et al., 2011).

3 This is indeed an oversimplification: we are aware the water market is much more complex than this. However, the point here is only to disentangle the service water purification from the benefit of cleaned water.

4 Ref. http://ec.europa.eu/eurostat/data/database.

5 There might be the possibility to value cleaned water in monetary terms, but in this case attention should be paid not to double count when the water purification service is already valued.

6 Reference macro-regions are: Northern Europe (Denmark), Southern Europe (Italy), Central Europe (Germany) and Eastern Europe (Bulgaria).

7 Ref. http://ec.europa.eu/environment/pubs/factsheets.htm.

8 Specifically from the Eurostat website: values at constant prices (2005 = 100) [aact_eaa03].

9 Considering only agriculture, one could add pollination, erosion control, global climate regulation, agro-biomass growing, animal husbandry, outdoor recreation. Considering the whole primary sector we would also include tree biomass growth and fish biomass maintenance.

10 Primary sector includes agriculture, forestry and fisheries. Data used for Bulgaria are not in constant prices, but in current prices.
